# Beamforming Design for Cooperative Intelligent Reflecting Surface-Assisted mmWave Communication

**DOI:** 10.3390/s22166214

**Published:** 2022-08-18

**Authors:** Yuyan Qian, Honggui Deng, Aimin Guo, Haoqi Xiao, Chengzuo Peng, Yinhao Zhang

**Affiliations:** School of Physics and Electronics, Central South University, Lushan South Road, Changsha 410083, China

**Keywords:** Intelligent Reflecting Surface, cooperative passive beamforming, IRS deployment, millimeter-wave communication, fractional programming

## Abstract

In order to investigate the effect of cooperative Intelligent Reflecting Surface (IRS) in improving spectral efficiency, this paper explores the joint design of active and passive beamforming based on a double IRS-assisted model. First, considering the maximum power constraint of the active vector and the unit modulus constraint of the cooperative passive vector, we establish the non-linear and non-convex optimization problem of multi-user maximization weighted sum rate (WSR). Then, we propose an alternating optimization (AO) algorithm to design the active vector and the cooperative passive vector based on fractional programming (FP) and successive convex approximations (SCA). In addition, we conduct a study on the optimization of the passive reflection vector under discrete phase shift. The simulation results show that the proposed beamforming scheme of double IRS-assisted model performs better than the conventional single IRS-assisted model.

## 1. Introduction

6G will provide all-around wireless coverage and interconnection of all things [1]. Intelligent Reflecting Surface (IRS) is one of the key technologies for 6G, which has the advantages of low energy consumption and simple deployment [2,3,4]. Specifically, IRS is a planar surface comprising a large number of reconfigurable passive electrical components that can adjust the phase shift of incident signal according to different channel conditions, leading to more reliable communication links, greater transmission capacity and higher spectral and energy efficiency [5,6].

Owing to these advantages, IRSs have attracted intensive attention and have been applied in several scenarios, including IRS-assisted integrated satellite unmanned aerial vehicle (UAV) terrestrial networks [7], IRS-assisted anti-jamming communications [8,9], cell-free massive multiple-input multiple-output (MIMO) system with IRS assistance [10] and IRS-assisted secure integrated terrestrial-aerial networks [11]. However, the deployment of IRS in different scenarios can greatly affect the performance and coverage of the system. To further improve the spectral efficiency and coverage of the system, how to jointly design beamforming according to the deployment scheme is a challenge to be solved.

Recently, most beamforming research is generally performed in single IRS-assisted systems [12,13]. In [12], the authors studied a single IRS-assisted multi-user downlink transmissions model under perfect and imperfect channel state information (CSI), respectively, and proposed a block coordinate descent (BCD) method to maximize the weighted sum rate (WSR). Furthermore, in [13], in the process of jointly active and passive vector optimization design, a low-complexity beamforming algorithm based on closed-form expressions of optimization variables was proposed. The aforementioned works mainly studied the single IRS-assisted models, which are not flexible, resulting in low coverage of wireless communication systems.

To further obtain higher spatial multiplexing gain and enhance coverage, the authors extended the scenario to distributed IRSs in [14], which broke the rank-one constraint of the base station (BS) to the IRS channel and developed a higher-rank channel, and they proposed an alternating optimization (AO) method to jointly design active and passive beamforming. Then, in [15], a hybrid active and passive wireless network with large-scale deployment of BSs and distributed IRSs was investigated to achieve network capacity growth by adjusting the optimal IRS to the BS density ratio.

In both [14,15], the distributed IRSs independently serve the related users in the local coverage area without considering the cooperation power between IRSs, which simplifies the beamforming design scheme. However, there will be signal reflections between IRSs in practice, and the role in a small range of environments is clear. The above passive beamforming scheme is no longer optimal when considering the inter-IRS reflection channel. Therefore, a new cooperative beamforming design is required.

To solve the signal interaction problem between cooperative IRSs, some recent works have investigated cooperative IRSs beamforming. In [16], the authors made a preliminary attempt to achieve far more than a conventional single IRS system by designing the passive beamforming of two cooperative IRSs; however, they only investigated the cooperative power gain of the double-reflection link without considering the spatial multiplexing gain brought by the two single-reflection links.

Moreover, in [17], the capacity maximization problem of MIMO system with double-IRS assistance was studied by jointly optimizing the transmit covariance matrix and the passive beamforming matrix under the line-of-sight (LoS) channel characteristics. However, the LoS path considered in [17] is an ideal model, and the application scenario is limited due to the failure to consider small-scale fading.

Then, the paper [18] considered double IRS-assisted the uplink communication system under the general channel conditions and adopted semidefinite relaxation (SDR) and the bisection method to effectively solve the problem of maximizing the minimum signal-to-interference-plus-noise ratio (SINR). However, the SDR method not only has a large number of iterations but also can only obtain approximate solutions because of Gaussian randomization. The results suggest that double-IRS model has a huge potential for improving spectral efficiency.

Inspired by the above works, we aim to maximize the WSR of the cooperative IRS-assisted millimeter wave (mmWave) downlink communication and propose a joint beamforming design scheme. The main contributions of this paper are as follows:

- First, for multivariate coupled non-convex optimization problems, we implement the decoupling of optimization variables based on fractional programming (FP) and convert the original problem equivalently into four subproblems in this paper.
- Next, we design an extended AO algorithm to solve the joint beamforming design problem under continuous phase shift to maximize the WSR of the system. In terms of the active transmission vector, quadratic transform (QT) is adopted to reconstruct the optimization problem and obtain a closed-form expression for the optimization variable. Regarding reflection vector optimization, the twice stochastic successive convex approximations (SCA) technique is adopted to find the optimal step size separately to achieve a joint optimization of the passive phase shifts.
- In addition, we extend the passive beamforming scheme to the case of discrete phase shifts of the IRS. The simulation results show that, for both continuous and discrete phase shifts, the proposed beamforming scheme of the double IRS-assisted system outperforms the conventional single IRS-assisted system.

The remainder of this paper is structured as follows. The double IRS-assisted system model and problem formulation are introduced in Section 2. In Section 3, we present the detailed derivations of the proposed low-complexity AO algorithm. The simulation results are analyzed in Section 4 to demonstrate the effectiveness of the proposed algorithm. Finally, we give our conclusions in Section 5.

The notations used in this paper are listed as follows. E{·} denotes statistical expectation. $CN\left(\mu ,\sigma_{0}^{2}\right)$ denotes the circularly symmetric complex Gaussian (CSCG) distribution with mean μ and variance $\sigma_{0}^{2}$. mim(a,b) denotes the minimum between two real numbers a and b. ⊗ denotes the Kronecker product. Superscripts $G^{*}$, $G^{T}$ and $G^{H}$ denote the conjugate, the transpose and the conjugate transpose of G, respectively. $C^{a\times b}$ denotes the space of a×b complex-valued matrices. For any vector ω, $\omega_{i}$ is the i-th element, ∥ω∥ and ${∥\omega ∥}_{F}$ denote the Euclidean norm and the Frobenius norm, respectively. Furthermore, diag(ω) denotes the diagonal matrix of vector ω. $I_{M}$ represents the M×M identity matrix. For any complex variable x, |x| denotes the absolute value of a complex variable x, and Re{x} represents the real part.

## 2. System Model and Problem Formulation

In this section, we present the transmission and channel model of a double IRS-assisted mmWave communication system and propose the problem formulation.

### 2.1. Transmission Model

**Figure 1:**
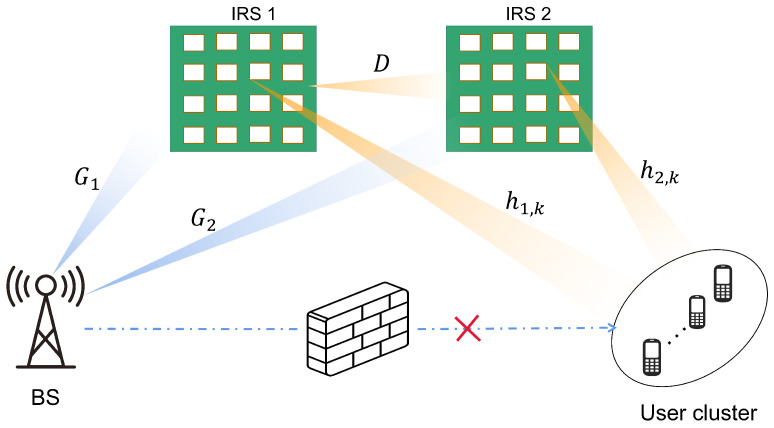
The cooperative double IRS-assisted multi-user MISO downlink communication system.

As shown in Figure 1, we consider a double IRS-assisted multi-user multiple-input single-output (MISO) downlink communication system, which consists of one BS equipped with *M* antennas and a cluster of *K* sigle-antenna users. Two cooperative IRSs are deployed in the network to provide high-quality transmission links. Considering the impact of path loss on the cooperative IRSs model, a realistic deployment scenario is developed in which IRS 1 and IRS 2 are placed near the user cluster and BS, respectively, to minimize the path loss.

To better represent the enhanced effect of IRS, we also consider that the direct links between the user equipment (UE) and the BS are blocked. Additionally, two IRSs are properly deployed to construct a LoS path from the IRSs to the BS and the users, so that the BS can effectively serve the users through the reflection link created by them.

To facilitate comparison with the single IRS deployment scheme, we assume that the total reflection elements of the single IRS and cooperative IRSs model consist of *N* elements, with IRS 1 and IRS 2 consisting of $N_{1}$ and $N_{2}$ reflection elements, respectively, with $N_{1}+N_{2}=N$. The corresponding phase shift matrix of IRS μ is denoted as $\Phi_{\mu }=diag\left(\theta_{\mu }\right),\mu \in \left\{1,2\right\}$, in which $\theta_{\mu }={\left[\begin{array}{c} \theta_{\mu ,1},\theta_{\mu ,2},\cdots ,\theta_{\mu ,N_{\mu }} \end{array}\right]}^{T}\in C^{N_{\mu }\times 1}$ denotes the equivalent reflection coefficient.

Then, the continuous phase feasible set can be expressed as $F_{c}=\left\{\theta_{\mu ,N_{\mu }}=e^{j\varphi_{\mu ,N_{\mu }}}|\varphi_{\mu ,N_{\mu }}\in \left[0,2\pi \right)\right\}$. In this case, the reflection amplitude of all elements is set to 1 to maximize the signal power and thus |θμ,n|=1. The feasible set corresponding to the discrete phase is given by $F_{d}=\left\{\theta_{\mu ,N_{\mu }}=e^{j\varphi_{\mu N_{\mu }}}|\varphi_{\mu ,N_{\mu }}\epsilon {\left\{\frac{2\pi i}{2^{B}}\right\}}_{i=0}^{2^{B}-1}\right\}$, where *B* is the phase resolution in the number of bits.

Each IRS consists of an arbitrary number of adjoining reflection elements, which, on the one hand, produce the same phase shift for the incident signal, bringing a high aperture gain, and on the other hand, their near-passive feature significantly reduces the cost of channel estimation and reflection optimization [19,20].

Let $G_{1}\in C^{N_{1}\times M}$, $G_{2}\in C^{N_{2}\times M}$, $D\in C^{N_{2}\times N_{1}}$, $h_{1,k}\in C^{N_{1}\times 1}$ and $h_{2,k}\in C^{N_{2}\times 1}$ denote the equivalent channels for BS→IRS1, BS→IRS2, IRS1→IRS2, IRS1→user, IRS2→user, respectively, where k={1,2,⋯,K}. Under the above conditions, the channels from BS to user *k* via a double-reflection link, and two single reflection links can be expressed as

(1) $$h_{k}^{H}=h_{1,k}^{H}\Phi_{1}G_{1}+h_{2,k}^{H}\Phi_{2}G_{2}+h_{2,k}^{H}\Phi_{2}D\Phi_{1}G_{1},$$

To highlight the fundamental performance gain of the double-IRS cooperative system, we assume that the CSI of all the above channels can be obtained accurately. In addition, we assume that all the channels are the quasi-static flat-fading channel model, which remains approximately constant within each channel coherence interval.

We set $s_{k}$ denote the transmitted signal from the BS to user *k* with $E{\left|s_{k}\right|}^{2}=1$, k={1,2,⋯,K}. Then, the transmitted signals for all users can be defined as

(2) $$x=\sum_{k=1}^{K}w_{k}s_{k},$$

where $w_{k}\in C^{M\times 1}$ is the corresponding transmit beamforming vector. During downlink data transmission, the signal received at user *k* can be expressed as

(3) $$y_{k}=\left(h_{1,k}^{H}\Phi_{1}G_{1}+h_{2,k}^{H}\Phi_{2}G_{2}+h_{2,k}^{H}\Phi_{2}D\Phi_{1}G_{1}\right)x+n_{k},$$

where $n_{k}∼\mathrm{CN}\left(0,\sigma_{0}^{2}\right)$. The *k*-th user is subject to interference from other users; thus, the corresponding SINR can be designated as

(4) $$\gamma_{k}=\frac{{\left|\left(h_{1,k}^{H}\Phi_{1}G_{1}+h_{2,k}^{H}\Phi_{2}G_{2}+h_{2,k}^{H}\Phi_{2}D\Phi_{1}G_{1}\right)w_{k}\right|}^{2}}{\sum_{\begin{array}{c} i=1\\ i\neq k \end{array}}^{K}{\left|\left(h_{1,k}^{H}\Phi_{1}G_{1}+h_{2,k}^{H}\Phi_{2}G_{2}+h_{2,k}^{H}\Phi_{2}D\Phi_{1}G_{1}\right)w_{i}\right|}^{2}+\sigma_{0}^{2}},$$

In addition, the transmit power constraint at BS is modeled as

(5) $$\sum_{k=1}^{K}{∥w_{k}∥}^{2}⩽P_{t},$$

where $P_{t}$ represents the maximum transmit power, and $W=\left[w_{1},w_{2},\ldots ,w_{K}\right]\in C^{M\times K}$ denotes the overall beamforming matrix at the BS.

### 2.2. Channel Model

Due to the sparse scattering characteristics of mmWave channels, this paper uses the 3D Saleh–Valenzuela channel model [21,22], where the BS is equipped with a uniform linear array (ULA), and the two IRSs are uniform planar arrays (UPA). Then, $G_{u}$ and D can be expressed as

(6) $$G_{u}=\sum_{\ell =0}^{L_{p}}v^{\left(\ell \right)}a_{B}\left(\phi_{B}^{\left(\ell \right)}\right)a_{u}^{H}\left(\phi_{u}^{\left(\ell \right)},\theta_{u}^{\left(\ell \right)}\right),$$

(7) $$D=\sum_{\ell =0}^{L_{d}}v^{\left(\ell \right)}a_{r}\left(\phi_{r}^{\left(\ell \right)},\theta_{r}^{\left(\ell \right)}\right)a_{t}^{H}\left(\phi_{t}^{\left(\ell \right)},\theta_{t}^{\left(\ell \right)}\right),$$

where $L_{p}$ and $L_{d}$ represent the number of non-line-of-sight (NLoS) paths, ℓ=0 denotes the LoS path, and $v^{\left(\ell \right)}$ is the complex gain of the *ℓ*-th path. The mmWave channels typically contain only a small number of major multipath components. Here, the azimuth and elevation angles are denoted by $\phi^{\left(\ell \right)}$ and $\theta^{\left(\ell \right)}$, a(ϕ,θ) denotes the array steering vector of IRS, and the array steering vector of the BS is expressed as

(8) $$a_{B}\left(\phi \right)=\frac{1}{\sqrt{M}}{\left[1,e^{j\frac{2\pi }{\lambda_{0}}d\sin\left(\varphi \right)},\ldots .e^{j\left(M-1\right)\frac{2\pi }{\lambda_{0}}d\sin\left(\varphi \right)}\right]}^{T},$$

where $\lambda_{0}$ is the signal wavelength, and $d=\frac{\lambda_{0}}{2}$ denotes the spacing between the antennas. It is assumed that each IRS unit has $N_{az}$ elements horizontally and $N_{el}$ elements vertically. Then, the array steering vector of IRS is expressed as

(9) $$a\left(\phi ,\theta \right)=a^{az}\left(\phi \right)⊗a^{el}\left(\theta \right),$$

where $a^{az}\left(\phi \right)$ and $a^{el}\left(\theta \right)$ are defined in the same manner as $a_{B}\left(\phi \right)$.

Typically, because the IRS is widely distributed in hotspot locations, the probability of LoS paths is high. The IRS-user channel model ignores the transmit power of more than two reflections due to suffering from severe path loss and only considers the LoS path [9]. Thus, the channel between the IRS and the *k*-th user is modeled by

(10) $$h_{u,k}=\sqrt{N_{u}}v_{k}\varrho_{r}\varrho_{t}a_{u}\left(\phi_{u},\theta_{u}\right),$$

where the $\varrho_{r}$ and $\varrho_{t}$ are the transmit and receive antenna gains, $v_{k}$ is the channel gain.

### 2.3. Proposed Problem Formulation

In this paper, we aim to maximize the WSR at the downlink transmission by jointly optimizing the active and the cooperative passive vector, subject to maximum transmit power and phase shift constraints. The optimization problem is formulated as

(11) $$\begin{array}{cc} (P_{1})\; & \max_{W,\theta_{1},\theta_{2}}f_{1}\left(W,\theta_{1},\theta_{2}\right)=\sum_{k=1}^{K}\omega_{k}\log\left(1+\gamma_{k}\right)\\ \; & s.t.|\theta \mu ,n|=1,\forall n=1,\ldots ,N_{u},\mu \in \left\{1,2\right\},\\ \; & \;\sum_{k=1}^{K}{∥w_{k}∥}^{2}⩽P_{t}, \end{array}$$

where the weight $\omega_{k}$ represents the priority of the *k*-th user. Since the non-convexity and non-linearity of problem $\left(P_{1}\right)$, it is difficult to solve it optimally. In addition, the optimization variables $\theta_{1}$, $\theta_{2}$ and W are deeply coupled; therefore, finding the global optimal solution is a challenge.

Problem $\left(P_{1}\right)$ is to maximize a sum-of-log-of-ratio objective, which is a typical FP problem [23]. To solve the multi-variate coupled problem, we first transform $\left(P_{1}\right)$ into a much low-complex problem by introducing optimization variable. It can be rewritten as

(12) $$\begin{array}{cc} (P_{2})\; & \max_{\alpha ,W,\theta_{1},\theta_{2}}f_{2}\left(\alpha ,W,\theta_{1},\theta_{2}\right)\\ \; & s.t.|\theta \mu ,n|=1,\forall n=1,\ldots ,N_{u},\mu \in \left\{1,2\right\},\\ \; & \;\sum_{k=1}^{K}{∥w_{k}∥}^{2}\leq P_{t},\\ \; & \;\alpha_{k}\geq 0,k=1,2,\ldots ,K, \end{array}$$

where

(13) $$\begin{array}{c} f_{2}\left(\alpha ,W,\theta_{1},\theta_{2}\right)=\sum_{k=1}^{K}\omega_{k}\log\left(1+\alpha_{k}\right)-\sum_{k=1}^{K}\omega_{k}\alpha_{k}+\sum_{k=1}^{K}\frac{\omega_{k}\left(1+\alpha_{k}\right)\gamma_{k}}{1+\gamma_{k}}, \end{array}$$

The parameters $\alpha_{1},\alpha_{2},\ldots \ldots ,\alpha_{K}$ are the auxiliary variables introduced for each user by the Lagrangian dual transform [24], and then $\alpha ={\left[\alpha_{1},\alpha_{2},\ldots ,\alpha_{K}\right]}^{T}$ is the auxiliary vector.

For ease of reading, Table 1 summarizes the main symbol notations used in this paper and their physical meanings.

## 3. Joint Active and Passive Beamforming

The above-mentioned original problem is transformed into an optimization problem containing α, W, $\theta_{1}$ and $\theta_{2}$. To solve the problem efficiently, we first, decouple the problem $\left(P_{2}\right)$ into four disjoint optimization subproblems. Then, we propose an extended low-complecity AO algorithm. In other words, we optimize the beamforming of the transmission vector and the cooperative reflection vector in an alternating iterative form. In addition, we choose the traditional single IRS-assisted scheme as the benchmark for performance analysis comparison. The details on how to optimize the variables alternately are given as follows.

First, we aim to find the optimal closed-form expression for α with given W, $\theta_{1}$ and $\theta_{2}$. Specifically, when fixed W, $\theta_{1}$ and $\theta_{2}$, the problem $\left(P_{2}\right)$ can be viewed as an unconstrained optimization problem for $\alpha_{k}$.

**Proposition** **1.** *When W, $\theta_{1}$ and $\theta_{2}$ are fixed, the optimal $\alpha_{k}$ can be obtained as*

(14) $$\alpha_{k}=\gamma_{k},$$

**Proof.**  Taking the partial derivative of $f_{2}\left(\alpha ,W,\theta_{1},\theta_{2}\right)$ with respect to $\alpha_{k}$ yields

(15) $$\frac{\partial f_{2}\left(\alpha ,W,\theta_{1},\theta_{2}\right)}{\partial \alpha_{k}}=\frac{\gamma_{k}-\alpha_{k}}{\left(1+\gamma_{k}\right)\left(1+\alpha_{k}\right)},$$

Let $\frac{\partial f_{2}\left(\alpha ,W,\theta_{1},\theta_{2}\right)}{\partial \alpha_{k}}=0$, we can obtain that $\alpha_{k}=\gamma_{k}$ makes the objective function $f_{2}\left(\alpha ,W,\theta_{1},\theta_{2}\right)$ maximized. As $\frac{\partial f_{2}\left(\alpha ,W,\theta_{1},\theta_{2}\right)}{\partial \alpha_{k}}>0$ when $\alpha_{k}<\gamma_{k}$, $\frac{\partial f_{2}\left(\alpha ,W,\theta_{1},\theta_{2}\right)}{\partial \alpha_{k}}<0$ when $\alpha_{k}>\gamma_{k}$.   □

Based on the obtained closed-form expression about $\alpha_{k}$, we propose solutions for the active vector W and the cooperative passive vector $\theta_{1}$ and $\theta_{2}$ in the next two subsections, respectively.

### 3.1. Active Beamforming Scheme

When α is fixed, we can formulate a new optimization problem with respect to the active vector W and the cooperative passive vector $\theta_{1}$ and $\theta_{2}$, which can be rewritten as

(16) $$\begin{array}{cc} \left(P_{3}\right)\; & \max_{W,\theta_{1},\theta_{2}}f_{3}\left(W,\theta_{1},\theta_{2}\right)=\sum_{k=1}^{K}\frac{{\bar{\alpha }}_{k}\gamma_{k}}{1+\gamma_{k}}\\ \; & s.t.|\theta \mu ,n|=1,\forall n=1,\ldots ,N_{u},\mu \in \left\{1,2\right\},\\ \; & \;\sum_{k=1}^{K}{∥w_{k}∥}^{2}\leq P_{t}, \end{array}$$

where ${\bar{a}}_{k}=\omega_{k}\left(1+\alpha_{k}\right)$, and the $\gamma_{k}$ can be rewritten as

(17) $$\gamma_{k}=\frac{{\left|h_{k}^{H}w_{k}\right|}^{2}}{\sum_{i=1,i\neq k}^{K}{\left|h_{k}^{H}w_{i}\right|}^{2}+\sigma_{0}^{2}},$$

when α, $\theta_{1}$ and $\theta_{2}$ are fixed, we ignore irrelevant variables and substitute (17) into (16), the problem $\left(P_{3}\right)$ can be formulated as

(18) $$\begin{array}{cc} (P_{4a})\; & \max_{W}f_{4a}\left(W\right)=\sum_{k=1}^{K}\frac{{\bar{a}}_{k}{\left|h_{k}^{H}w_{k}\right|}^{2}}{\sum_{i=1}^{K}{\left|h_{k}^{H}w_{i}\right|}^{2}+\sigma_{0}^{2}}\\ \; & s.t.\sum_{k=1}^{K}{∥w_{k}∥}^{2}\leq P_{t}, \end{array}$$

The above problem is a multi-ratio sum problem with respect to W, and it can be reformulated as a biconvex optimization problem by QT [24]. Thus, the objective function can be reformulated as

(19) f4b(W,β)=∑k=1K2α¯kReβk*hkHwk−∑k=1Kβk2∑i=1KhkHwi2+σ02,

where $\beta_{1},\beta_{2},\cdots ,\beta_{K}$ are the introduced complex auxiliary variables, and $\beta ={\left[\beta_{1},\beta_{2},\cdots ,\beta_{K}\right]}^{T}$ is the auxiliary vector. Problem $\left(P_{4a}\right)$ is therefore equated to solving the biconvex problem with respect to β and W as follows

(20) $$\begin{array}{cc} (P_{4b})\; & \max_{W,\beta }f_{4b}\left(W,\beta \right)\\ \; & s.t.\sum_{k=1}^{K}{∥w_{k}∥}^{2}\leq P_{t}, \end{array}$$

**Proposition** **2.** *When W is fixed, the optimal $\beta_{k}$ can be updated by*

(21) $$\beta_{k}=\frac{\sqrt{{\bar{\alpha }}_{k}}h_{k}^{H}w_{k}}{\sum_{i=1}^{K}{\left|h_{k}^{H}w_{i}\right|}^{2}+\sigma_{0}^{2}},$$

*Then, the optimal beamforming vector $w_{k}$ is obtained by the Lagrange multiplier method, which can be expressed as*

(22) $$w_{k}=\sqrt{{\bar{a}}_{k}}\beta_{k}{\left(\lambda I_{M}+\sum_{i=1}^{K}{\left|\beta_{i}\right|}^{2}h_{i}h_{i}^{H}\right)}^{-1}h_{k},$$

**Proof.**  When W is fixed, taking the partial derivative of $f_{4b}\left(W,\beta \right)$ with respect to $\beta_{k}$ yields

(23) $$\frac{\partial f_{4b}\left(W,\beta \right)}{\partial \beta_{k}}=2\sqrt{{\bar{\alpha }}_{k}}h_{k}^{H}w_{k}-2\beta_{k}\sum_{i=1}^{K}{\left|h_{k}^{H}w_{i}\right|}^{2}-2\beta_{k}\sigma_{0}^{2},$$

Let $\frac{\partial f_{4b}\left(W,\beta \right)}{\partial \beta_{k}}=0$, then the above Equation (21) is obtained. When β is fixed, considering the maximum transmit power constraint, the Lagrangian function of $f_{4b}\left(W,\beta \right)$ can be obtained as

(24) $$\begin{array}{cc} f_{4c}\left(\lambda ,W,\beta \right) & =\sum_{k=1}^{K}\sqrt{{\bar{\alpha }}_{k}}\left(\beta_{k}^{*}h_{k}^{H}w_{k}+w_{k}^{H}h_{k}\beta_{k}\right)\\ \; & -\sum_{i=1}^{K}{\left|\beta_{i}\right|}^{2}\sum_{k=1}^{K}w_{k}^{H}h_{i}h_{i}^{H}w_{k}-\lambda \left(\sum_{k=1}^{K}w_{k}^{H}w_{k}-P_{t}\right), \end{array}$$

where λ is the dual variable introduced by considering about the power constraint and the partial derivative of $f_{4c}\left(\lambda ,W,\beta \right)$ with respect to $w_{k}$ as follows

(25) $$\frac{\partial f_{4c}\left(\lambda ,W,\beta \right)}{\partial w_{k}}=2\sqrt{{\bar{\alpha }}_{k}}h_{k}\beta_{k}-2\sum_{i=1}^{K}{\left|\beta_{i}\right|}^{2}h_{i}h_{i}^{H}w_{k}-2\lambda I_{M}w_{k},$$

let $\frac{\partial f_{4c}\left(\lambda ,W,\beta \right)}{\partial w_{k}}=0$, the above expression (22) can be obtained.    □

In general, the optimal λ can be obtained using the bisection method, and a large number of iterations of searching is generally required to obtain a high-accurate W. However, to avoid operating the matrix inverse too many times, we take the following update form of W [12].

(26) W=argminW∑k=1KRegkHwk−w^k+L2wk−w^k2s.t.∑wk2≤Pt,

where L>0, the gradient is expressed as

(27) $$g_{k}=-{\left.\frac{\partial f_{4b}}{\partial w_{k}}\right|}_{w_{k}={\hat{w}}_{k}}=-2\sqrt{{\bar{a}}_{k}}\beta_{k}\;h_{k}+2\sum_{i=1}^{K}{\left|\beta_{i}\right|}^{2}\;h_{i}\;h_{i}^{H}{\hat{w}}_{k},$$

where ${\hat{w}}_{k}=w_{k}^{\left(l-1\right)}+\epsilon \left(w_{k}^{\left(l-1\right)}-{\ddot{w}}_{k}\right)$ is the extrapolation point, $w_{k}^{\left(l-1\right)}$ is the value of the last alternate iteration, ${\ddot{w}}_{k}$ is the value of $w_{k}$ before it was updated to $w_{k}^{\left(l-1\right)}$, and ϵ>0 denotes the extrapolation weight. The updated expression for $w_{k}$ is

(28) $$w_{k}=\frac{1}{L-2\lambda }\left(L{\hat{w}}_{k}-g_{k}\right),$$

(29) $$\lambda =\frac{L}{2}-\frac{1}{2P_{t}}\sum_{k=1}^{K}{∥L{\hat{w}}_{k}-g_{k}∥}^{2},$$

Using this linear approximation eliminates the need for a bisection search. We let (l−1) represent the value of the previous iteration and (l) is the value of the current iteration. $L=2{∥\sum_{i=1}^{K}{\left|\beta_{i}\right|}^{2}h_{i}h_{i}^{H}∥}_{F}$ is the Lipschitz constant of the gradient function $g_{k}$. Furthermore, the extrapolation weight is taken by

(30) $$\epsilon =\min\left(\frac{d^{\left(l\right)}-1}{d^{\left(l-1\right)}},0.9999\sqrt{\frac{L^{\left(l-1\right)}}{L^{\left(l\right)}}}\right),$$

where $d^{\left(l\right)}$ is defined as $d^{\left(l\right)}=\frac{1}{2}\left(1+\sqrt{1+4d^{{\left(l-1\right)}^{2}}}\right)$ with initial value 1.

### 3.2. Passive Beamforming Scheme

When optimizing cooperative reflection vector $\theta_{1}$, we first, fix α, W and $\theta_{2}$. Then, by denoting ${\tilde{D}}_{k}≜{\left[{\tilde{d}}_{k,1},\cdots ,{\tilde{d}}_{k,N_{2}}\right]}^{H}=diag\left(h_{2,k}^{H}\right)D\in C^{N_{2}\times N_{1}}$, we can transform the channel $h_{k}^{H}$ to be further expressed as

(31) $$\begin{array}{cc} h_{k}^{H} & =\theta_{2}^{H}{\tilde{D}}_{k}\Phi_{1}G_{1}+\theta_{2}^{H}T_{2,k}+\theta_{1}^{H}T_{1,k}\\ \; & =\theta_{2}^{H}{\left[{\tilde{d}}_{k,1}\Phi_{1},\ldots ,{\tilde{d}}_{k,N_{2}}\Phi_{1}\right]}^{H}G_{1}+\theta_{2}^{H}T_{2,k}+\theta_{1}^{H}T_{1,k}\\ \; & =\theta_{2}^{H}{\left[\theta_{1}^{H}diag\left({\tilde{d}}_{k,1}\right),\ldots ,\theta_{1}^{H}diag\left({\tilde{d}}_{k,N_{2}}\right)\right]}^{H}G_{1}+\theta_{2}^{H}T_{2,k}+\theta_{1}^{H}T_{1,k}\\ \; & =\sum_{n=1}^{N_{2}}\theta_{1}^{H}\theta_{2,n}\underset{T_{k,n}}{\underset{︸}{diag\left({\tilde{d}}_{k,n}\right)G_{1}}}+\theta_{2}^{H}T_{2,k}+\theta_{1}^{H}T_{1,k}, \end{array}$$

where $T_{1,k}=diag\left(h_{1,k}^{H}\right)G_{1}$ and $T_{2,k}=diag\left(h_{2,k}^{H}\right)G_{2}$ represent the channel components of the two single-reflection links, respectively. $T_{k,n}$ represents the channel components of the cooperative double-reflection link. Then, $f_{3}\left(W,\theta_{1},\theta_{2}\right)$ is further expressed as

(32) f5aθ1=∑k=1K2a¯kReβk*θ1HT1,k+θ2HT2,k+∑n=1N2θ1Hθ2,nTk,nwk−∑k=1Kβk2∑i=1Kθ1HT1,k+θ2HT2,k+∑n=1N2θ1Hθ2,nTk,nwi2+σ02,

Then, the sub-optimization problem with respect to $\theta_{1}$ can be transformed to be

(33) (P5)θ^1=argminθ1f5bθ1≜θ1HAθ1−2Reθ1HBs.t.θ1,n=1,∀n=1,⋯,N1,

where A and B are

(34) $$A=\sum_{k=1}^{K}{\left|\beta_{k}\right|}^{2}\sum_{i=1}^{K}a_{i,k}a_{i,k}^{H},$$

(35) $$B=\sum_{k=1}^{K}\left(\sqrt{{\bar{\alpha }}_{k}}\beta_{k}^{*}a_{k,k}-{\left|\beta_{k}\right|}^{2}\sum_{i=1}^{K}b_{i,k}^{*}a_{i,k}\right),$$

with $a_{i,k}=\left(T_{1,k}+\sum_{n=1}^{N_{2}}\theta_{2,n}T_{k,n}\right)w_{i}$ and $b_{i,k}=\left(\theta_{2}{}^{H}T_{2,k}\right)w_{i}$. We further replace $\theta_{1,n}$ by $\varphi_{1,n}$ with $\theta_{1,n}=e^{j\varphi_{1,n}}$. Then, we can obtain as follows

(36) φ1=argminφ1∈RNf5cφ1≜ejφ1HAejφ1−2ReBHejφ1

where $\varphi_{1}={\left[\varphi_{1,1},\cdots ,\varphi_{1,N_{1}}\right]}^{T}$; however, $f_{5c}\left(\varphi_{1}\right)$ is still non-convex. Thus, we further exploit the SCA technique to solve this problem, with the construction of surrogate function referring to [25]. We use the second order Taylor expansion at $\varphi_{1}^{\left(l-1\right)}$ to construct the surrogate function which can be expressed as

(37) $$\begin{array}{cc} f_{5d}\left(\varphi_{1},\varphi_{1}^{\left(l-1\right)}\right)\; & =f_{5c}\left(\varphi_{1}^{\left(l-1\right)}\right)+\nabla f_{5c}{\left(\varphi_{1}^{\left(l-1\right)}\right)}^{T}\left(\varphi_{1}-\varphi_{1}^{\left(l-1\right)}\right)\\ \; & +\frac{\kappa }{2}{∥\varphi_{1}-\varphi_{1}^{\left(l-1\right)}∥}^{2}, \end{array}$$

where ∇f5cφ1(l−1)=2Re−jθ1(l−1)*∘Aθ1(l−1)−B, and the optimization problem can be expressed as

(38) $$\varphi_{1}=\arg\min_{\varphi_{1}\in R^{N}}f_{5d}\left(\varphi_{1},\varphi_{1}^{\left(l-1\right)}\right),$$

The expression for the iteration with respect to $\varphi_{1}$ is given by

(39) $$\varphi_{1}=\varphi_{1}^{\left(l-1\right)}-\frac{\nabla f_{5c}\left(\varphi_{1}^{\left(l-1\right)}\right)}{\kappa },$$

In order to speed up the iteration of the algorithm, a proper search step size κ in (39) is needed. Furthermore, we consider optimization problem as follows

(40) P6minφ1f6aφ1≜ejφ1HAejφ1−2ReBHejφ1

To solve problem ($P_{6}$), we construct function as follows

(41) $$f_{6b}\left(\varphi_{1},\varphi_{1}^{\left(l-1\right)}\right)=f_{6a}\left(\varphi_{1}^{\left(l-1\right)}\right)+\nabla f_{5c}{\left(\varphi_{1}^{\left(l-1\right)}\right)}^{T}\left(\varphi_{1}-\varphi_{1}^{\left(l-1\right)}\right)+\frac{\kappa }{2}{∥\varphi_{1}-\varphi_{1}^{\left(l-1\right)}∥}^{2},$$

It is known that $f_{6a}$ is a continuously differentiable function, and the gradient of $f_{6a}$ is

(42) ∇f6aφ1=∇f5cφ1=2Re−je−jφ1∘Aejφ1−B,

The step size κ in this paper can be determined by the Armijo rule [26].

(43) $$f_{6a}\left(\varphi_{1}^{\left(l-1\right)}\right)-f_{6a}\left(\varphi_{1}\right)\geq \zeta \kappa {∥\nabla f_{5c}\left(\varphi_{1}^{\left(l-1\right)}\right)∥}_{2}^{2},$$

where 0<ζ<0.5, κ is the largest element in the ${\left\{\kappa_{0}^{-j}\right\}}_{j=0,1,\ldots }$ and $\kappa_{0}>1$.

Next, we optimize the reflection vector $\theta_{2}$ of IRS 2, meanwhile the remaining variables are fixed. The problem $\left(P_{3}\right)$ can be rewritten as

(44) (P7)θ^2=argminθ2f7aθ2≜θ2HJθ2−2Reθ2HZs.t.θ2,n=1,∀n=1,⋯,N2,

we construct J and Z as follows

(45) $$J=\sum_{k=1}^{K}{\left|\beta_{k}\right|}^{2}\sum_{i=1}^{K}q_{i,k}q_{i,k}^{H},$$

(46) $$Z=\sum_{k=1}^{K}\left(\sqrt{{\bar{\alpha }}_{k}}\beta_{k}^{*}q_{k,k}-{\left|\beta_{k}\right|}^{2}\sum_{i=1}^{K}p_{i,k}^{*}q_{i,k}\right),$$

where $q_{i,k}=\left(T_{2,k}+diag\left(h_{2,k}^{H}\right)D\Phi_{1}G_{1}\right)w_{i}$ and $p_{i,k}=\left(\theta_{1}{}^{H}T_{1,k}\right)w_{i}$. Following the similar transformations from (36) to (43), we can again obtain the optimal reflection vector $\theta_{2}$ of IRS 2 by determining the appropriate step size based on the Armijo rule.

In the proposed AO algorithm, we transform the original problem into four subproblems, and the beamforming algorithm design accommodated to the cooperative double IRS-assisted model is implemented by iteratively solving the four subproblems. In particular, the solution obtained in each iteration is used as the initial point for the next iteration. The details of the proposed algorithm to achieve WSR maximization are summarized in Algorithm 1.
**Algorithm 1:** The AO Algorithm for joint design of active and cooperative passive beamforming.1. Initialization: Set feasible values of $\left\{w^{0},\theta_{1}^{0},\theta_{2}^{0}\right\}$. Set the threshold value τ and the maximum mumber of iterations $I_{m}$. Iteration i=0.2. Repeat3. Update $a^{i}$ according to (14).4. Update $\beta^{i}$ according to (21).5. Update $W^{i}$ according to (28) and (29).6. Construct **A** and **B** by (34) and (35).7. Update $\theta_{1}^{i}$ according to (39).8. With given $\theta_{1}^{i}$, update $\Phi_{1}^{i}$.9. Construct **J** and **Z** by (45) and (46).10. Update $\theta_{2}^{i}$.11. With given $\theta_{2}^{i}$, update $\Phi_{2}^{i}$.12. Set i=i+1.13. Until the increase of the WSR value is below the threshold τ or the interation number i reaches the $I_{m}$.14. Perform the quantized phase projection.

## 4. Simulation Results

**Figure 2:**
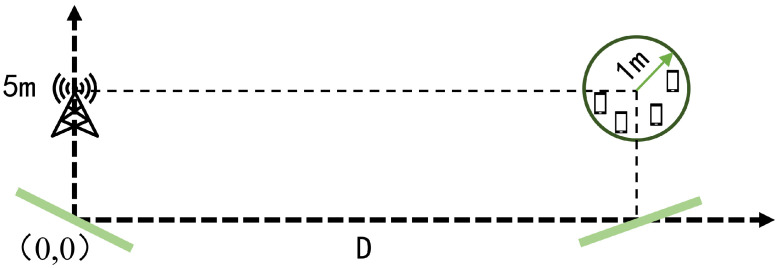
The simulated cooperative IRS-assisted multi-user communications.

In this section, simulation results are used to examine the performance of the cooperative double IRS-assisted multi-user system and to verify the feasibility of AO algorithm. The considered system is depicted in Figure 2, in which one BS is equipped with 4 antennas and 4 single-antenna users (K=4) randomly distributed in a circle center at (50m,5m) with a radius of 1 m. Since the direct link channel from the BS to the UE is not considered, our weight value is chosen as $\omega_{k}=\frac{1}{K}$.

According to [21], the channel gain is taken as $v_{k}∼CN\left(0,10^{-0.1PL(r)}\right)$ where $PL\left(r\right)=\varrho_{a}+10\varrho_{b}\mathrm{lg}\left(r\right)+\xi$ with $\xi ∼N{\left(0,\sigma_{\xi }^{2}\right)}^{3}$. The channel realizations are produced by setting $\sigma_{0}^{2}=-85\;\mathrm{dBm}$, $\varrho_{t}=9.82\;\mathrm{dBi}$, $\varrho_{r}=0\;\mathrm{dBi}$, $\varrho_{a}=61.4$, $\varrho_{b}=2$, $\sigma_{\xi }=5.8\;\mathrm{dB}$. The BS is located at (0 and 5 m), and two IRSs with the number of elements $N_{1}=N_{2}=30$ are located at (0m,0m) and (50m,0m), respectively. Furthermore, we consider the transmit power of the BS is set to $P_{t}=30\;\mathrm{dBm}$, and the number of quantized bits is B=3.

We focus on the impact of the main system parameters on the WSR, adopting three benchmarks to evaluate the performance of the model and algorithm: (1) cooperative IRS phase randomization, and then W is optimized using the weighted minimum mean square error (WMMSE) [27] (Random Phase); (2) single IRS scheme near the BS; and (3) single IRS scheme near the UE.

**Figure 3:**
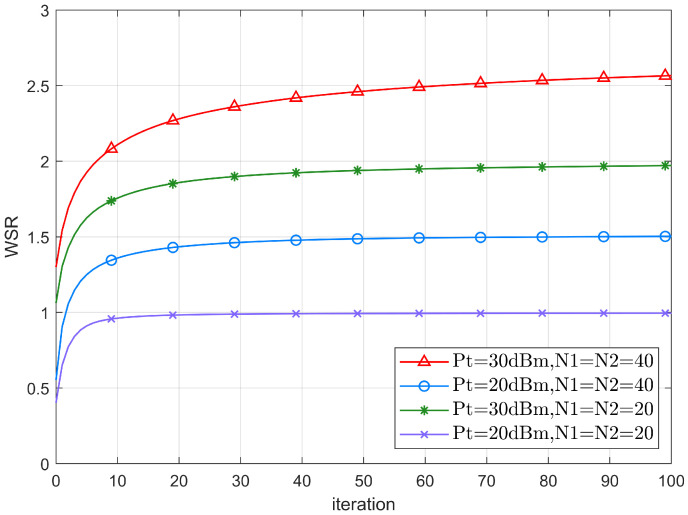
The convergence behavior of the AO algorithm.

Figure 3 shows the WSR convergence behaviors of the system for different transmit power and the number of IRS reflection elements. It can be seen that for different $P_{t}$ and reflection elements, the WSR gradually converges to a smooth level as the number of iterations increases, proving the practicality of the proposed algorithm. In particular, at the transmit power of 20dBm, the WSR converges in about 20 iterations when the number of two IRSs reflection elements is 20.

Still, when the number of two IRSs reflection elements is increased to 40, it takes 40 iterations before the WSR converges. This is because as the number of reflection elements gradually increases, the number of variables to be optimized increases, resulting in a decrease in the convergence rate. As the transmit power increases, the SINR increases and better performance of the system is obtained, and thus the number of iteration steps required to achieve a higher convergence value increases accordingly.

**Figure 4:**
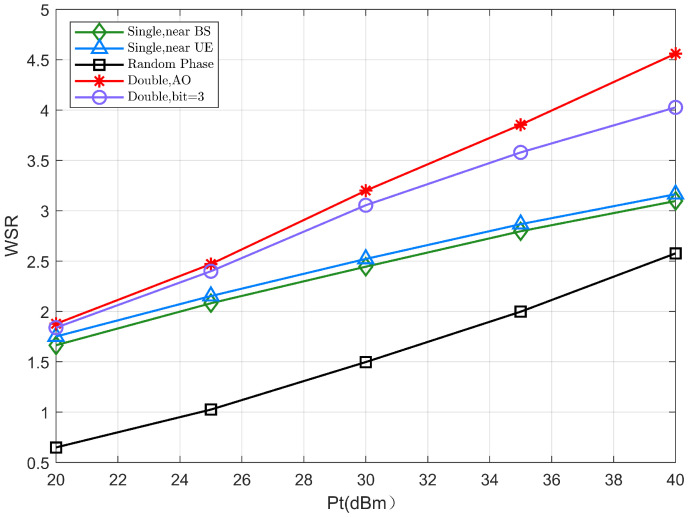
The WSR versus the transmit power $P_{t}$.

In Figure 4, we fix the number of reflection elements of the two IRSs to be $N_{1}=N_{2}=30$, demonstrating the variation in performance of the WSR with varying transmit power $P_{t}$. It can be seen that the double-IRS model shows better performance than the single IRS (N=60) model for both continuous and discrete phase shift scenarios. Especially, considering a reference value at $P_{t}=30\;\mathrm{dBm}$, the joint beamforming based on the double-IRS scheme can achieve about 3dB gain compared to the single IRS scheme.

This is mainly because the cooperative double-IRS system has the spatial multiplexing gain of the two single-reflection links in addition to the cooperative power gain of the double-reflection link, which helps to improve the WSR of the system. In addition, the WSR in the single IRS scheme is not sensitive enough to the transmit power, the cooperative double IRS scheme has an increasingly faster performance improvement in case of the transmit power gradually increases, and the difference between them is more pronounced as the transmit power increases.

**Figure 5:**
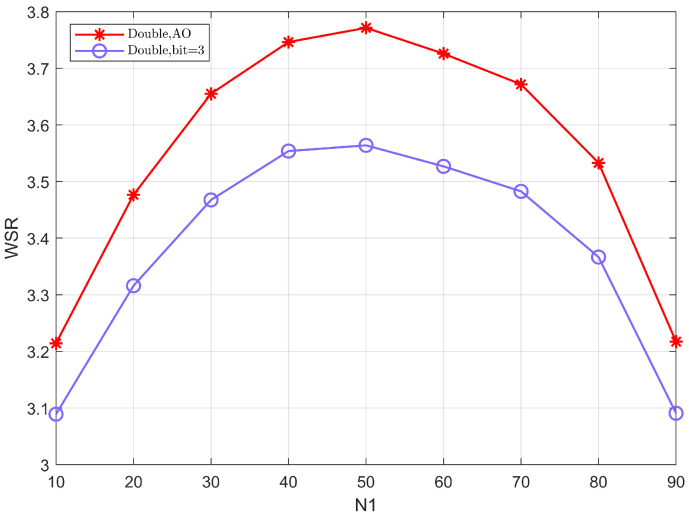
The WSR versus the number of the reflection elements $N_{1}$ assigned to IRS 1.

Figure 5 shows the WSR versus the number of the reflection elements $N_{1}$ assigned to IRS 1 when other conditions are the same and we fix the total number of elements to be $N=N_{1}+N_{2}=100$. From the figure, it can be seen that the value of WSR is greatest when the number of elements of the two cooperative IRSs is equal. This is primarily because reasonably assigned elements can efficiently balance the passive beamforming gain, resulting in improved system performance.

**Figure 6:**
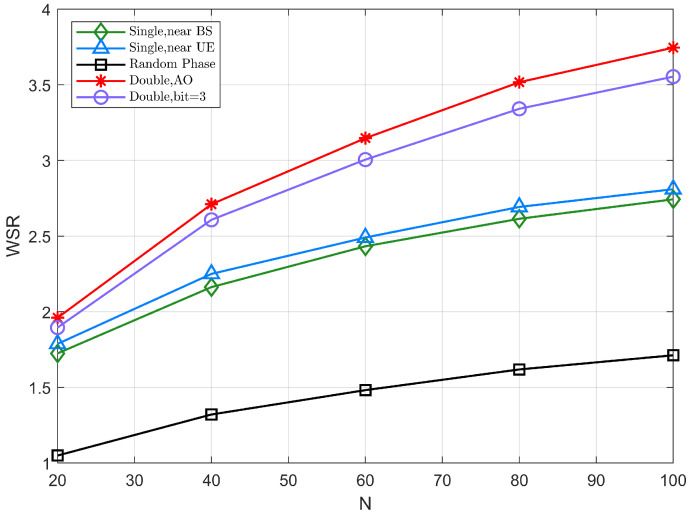
The WSR versus the total number of the reflection elements *N*.

Figure 6 illustrates the WSR of different schemes with respect to the number of reflection elements N when $P_{t}=30\;\mathrm{dBm}$, where $N_{1}=N_{2}=\frac{N}{2}$. The WSR gradually increases with the number of reflection elements in each of the different scenarios, as the increase in the number of reflection elements improves the array gain of the IRS. Among them, the cooperative IRS model significantly improves spectral efficiency than the single IRS model.

Considering a reference value at N=60, the joint beamforming based on the double-IRS scheme can achieve about 3dB gain compared with a single IRS scheme, because the cooperative system obtains better channel rank conditions by adopting the double-IRS deployment. In addition, while the growth of WSR in the single IRS model varies smoothly with the number of reflection elements, the double-IRS model improves its performance at an increasing rate with the increase in the number of reflection elements, consistently maintaining superior system performance. Furthermore, their difference is more obvious as the number of reflection elements increases.

**Figure 7:**
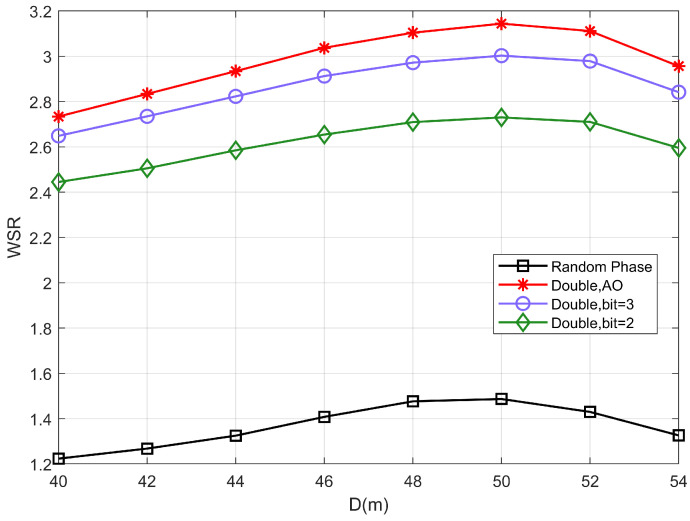
The WSR versus the horizontal coordinate of IRS 2.

Figure 7 shows the relationship between the WSR of the system as IRS 2 is moved from (40m,0m) to (54m,0m). D represents the horizontal coordinate of IRS 2 i.e., the horizontal distance between two IRSs. It depicts that when D increases from 50m to 54m, the system performance gradually decreases as the path loss of channel D becomes larger. Moreover, the WSR increases as the IRS 2 moves from (40m,0m) to (50m,0m), indicating that it is not the case that the closer the distance between the two IRSs is the better.

This is because the decrease in the inter-IRS distance decreases the path loss of the channel D; however, along with the change in D, the distance between the user and the IRS 2 also changes. In addition, the reduction of the sum of transmission distances does not necessarily mean that the transmission conditions become better. Therefore, when deploying locations of IRSs, it is necessary to consider not only the factor of path loss, but also the location of the users.

**Figure 8:**
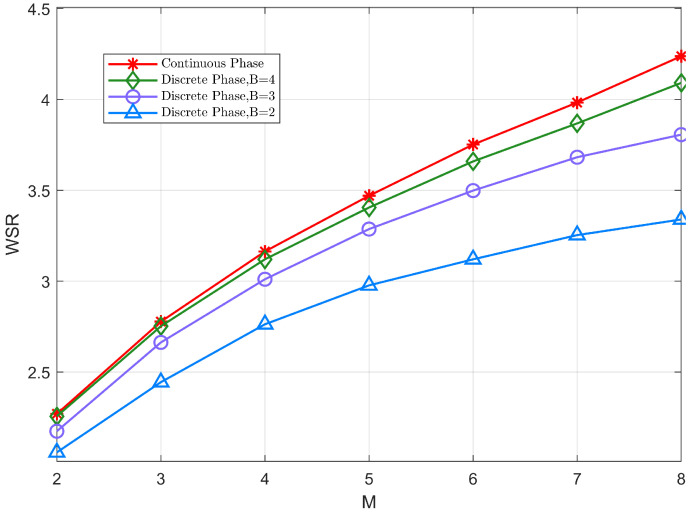
The WSR versus the number of transmit antennas M at the BS.

Figure 8 shows the variation of WSR with the number of antennas at the BS for both discrete and continuous schemes. According to the figure, in the discrete phase shift case, the larger the number of quantized bits (B = 4), the closer the system WSR is to the continuous case. This is because the performance loss caused by the rounding process during quantization is smaller at higher bit counts. It also shows that the proposed approach is suitable for discrete phase shifter with a high level of phase shift. However, the implementation difficulty increases as the number of quantized bits increases in practice. Thus, we only need to use a small number of quantized bits (B = 3) to achieve satisfactory system performance.

**Figure 9:**
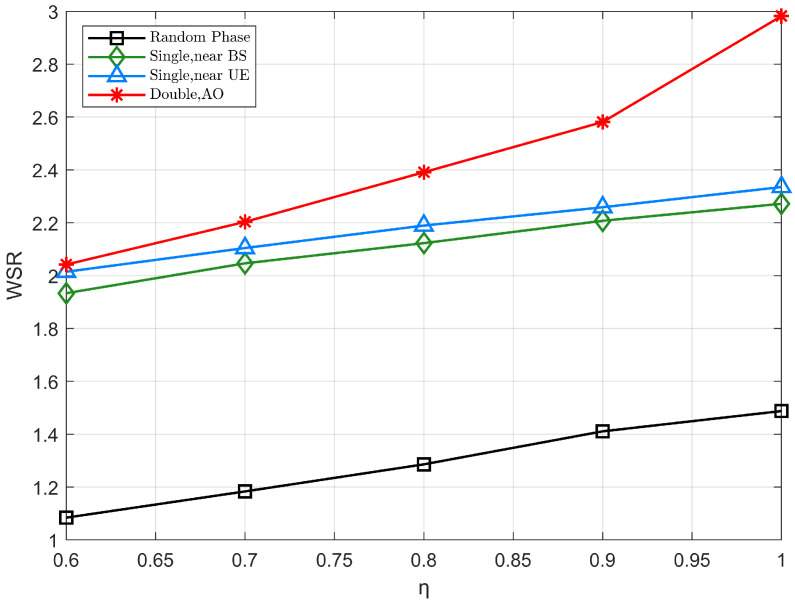
The WSR versus the reflection amplitude η.

Considering the practical application, there may be power loss at the IRS due to the absorption reflection of the phase shifter of the IRS, which we define as η with $\Phi_{\mu }≜\eta \mathrm{diag}\left(\theta_{\mu }\right),\mu \in \left\{1,2\right\}$. Figure 9 investigates the effect of η on the WSR of the system, and as the power loss decreases, the WSR of the system increases significantly. Specifically, the enhancement effect of cooperative IRS scheme is more obvious when η is close to 1. Furthermore, when η increases from 0.6 to 1, the WSR based on the double IRS-assisted scheme achieves about 4 dB gain.

## 5. Conclusions

This paper investigated a model for cooperative IRS-assisted downlink mmWave MISO communication, where the IRS-assisted effect is achieved through one double-reflection link and two single-reflection links. The WSR of the system is maximized by jointly designing the transmit vector and cooperative IRS phase under the constraints of the transmit power and unit modulus constraint. We investigated the performance advantages of the double-IRS model under continuous and discrete phase shift conditions, respectively.

To solve the non-convex and non-linear problem, the original problem was, first, simplified based on FP and then solved by exploiting the SCA technique. Finally, a beamforming method based on alternating iterations was designed to accommodate the cooperative double IRS-assisted model. The simulations showed that the double IRS-assisted scheme exhibited higher performance through cooperative reflection beamforming compared with the conventional single IRS-assisted scheme. In future work, it will be necessary to investigate multi-hop IRS-assisted systems and design beamforming.

## Figures and Tables

**Table 1:** Symbols and their physical meanings.

Symbols	Physical Meanings
$\lambda_{0}$	Signal wavelength
$\sigma_{0}^{2}$	Average noise power
*B*	The phase resolution in the number of bits
$a_{B}$	The array steering vector of the BS
$a_{u}$/$a_{r}$/ $a_{t}$	The array steering vector of the IRS
κ	Search step size
ϵ	Extrapolation weight
(l)/(l−1)	The value of the current/previous iteration

## Data Availability

Not applicable.
